# Telenutrition for Inflammatory Bowel Disease: A Tipping Point for Dietary Wellness

**DOI:** 10.1093/crocol/otab017

**Published:** 2021-04-15

**Authors:** Sami Elamin, Jonah Cohen

**Affiliations:** Hospital Medicine Unit, Department of General Internal Medicine, Massachusetts General Hospital, Boston, Massachusetts, USA; Harvard Medical School, Boston, Massachusetts, USA; Harvard Medical School, Boston, Massachusetts, USA; Center for Advanced Endoscopy, Beth Israel Deaconess Medical Center, Boston, Massachusetts, USA

**Keywords:** telehealth, telenutrition, inflammatory bowel disease, diet, dietary wellness, Crohn’s disease, ulcerative colitis, telemedicine, nutrimedy

## Abstract

Inflammatory bowel disease (IBD), Crohn’s disease and ulcerative colitis, cause inflammation of the digestive tract. It is estimated that about three million Americans and, globally, over six million individuals, suffer from IBD. While most physicians, especially gastroenterologists, are experts in the function and pathology of the gastrointestinal tract, factors such as nutrition science education and training, bandwidth, culture, language, and the longitudinal nature of dietary care, represent some of the barriers to receiving optimal nutritional guidance. Remote dietary expert counseling, an emerging solution that has been further highlighted by the COVID-19 pandemic, can improve IBD patients’ nutritional status, avoid food triggers, and reduce the frequency and severity of exacerbations.

## INTRODUCTION

Diet plays an essential role in the prevention and management of chronic disease. Healthier eating is increasingly being recognized as a cornerstone of wellness in the United States and globally.^1^ As a chronic autoimmune condition, inflammatory bowel disease (IBD) is comprised of 2 major disorders: Crohn’s disease (CD) which is characterized by transmural inflammation that can involve any portion of the gastrointestinal tract, and ulcerative colitis (UC) which causes inflammation of the mucosal layer of the colon. The age of onset for most patients with UC and CD is between 15 and 30 years, however IBD can present at any age and evidence suggests a bimodal distribution with elderly onset in many.^2,3^ Diarrhea, rectal bleeding, abdominal pain, and fatigue are often the primary symptoms at presentation. In CD, complications include but are not limited to development of strictures, fistulas, abscesses, perianal disease, and malabsorption. IBD patients may also suffer from extra intestinal manifestations involving the eyes, skin, and joints.^4,5^ Although advances in medical therapy have been made, many patients with IBD, especially CD, may require surgical intervention.^6^ In addition to having a higher risk of developing colon cancer,^7–11^ IBD patients have a higher mortality rate than the general population.^12^

## NUTRITION IN IBD

The cardinal symptoms of IBD, namely abdominal pain, nausea, and diarrhea can result in poor appetite, reduced oral intake and ultimately, together with malabsorption can lead to impaired nutritional status. This manifests in many forms including weight loss, growth failure, reduced muscle, and bone mass, as well as micronutrient deficiencies. Besides avoiding trigger foods that exacerbate symptoms, dietary interventions in IBD can help patients maintain adequate oral intake to avoid malnutrition and optimize their nutritional status.

A detailed history is critical in identifying IBD patients at risk of undernutrition and must include questions regarding altered taste, appetite, and activity level. A thorough physical examination should take into account loss of subcutaneous fat and/or muscle mass as well as evaluation of handgrip strength which, if decreased, may be an indication of poor nutritional status.^13^ The Subjective Global Assessment score (SGA) can be used to identify patients with, or at risk of developing undernutrition.^14^ An array of laboratory tests exist to monitor disease activity and nutritional status in patients with IBD including but not limited to complete blood count (CBC), erythrocyte sedimentation rate (ESR), C-reactive protein (CRP), albumin, 25-hydroxyvitamin D, iron studies, calcium, phosphorus, magnesium, vitamins A/E/B12, prothrombin time/international normalized ratio (PT/INR), zinc, folate, and dual-energy X-ray absorptiometry (DXA) scanning.

While clinical studies in the area of nutrition and dietary management in IBD are small in number, data from several epidemiologic studies suggest that certain measures can improve nutritional status and avoid food triggers for the majority of affected patients. Chapman-Kiddell et al sought to evaluate the role played by environmental factors. They identified diet and the host microbiota as potentially being as responsible as genetic susceptibility for the increasing prevalence of IBD globally.

Upon examining the “Western” diet, mechanisms such as insulin resistance, modification of intestinal permeability, and the effect of sulfur compounds from protein have been identified as contributors to intestinal inflammation.^15^ For example, increased intake of polyunsaturated fatty acids and animal fat has been linked to an increased incidence of UC and CD, and relapse in patients with UC.^16–19^ Additionally, a lower intake of omega-6 fatty acids and a higher intake of omega-3 fatty acids have been associated with a lower risk of developing CD.^20^ Vitamin D deficiency is common in patients with IBD, and an inverse relation exists between vitamin D intake and the risk for developing CD.^21,22^ Furthermore, consumption of fruits and cruciferous vegetables containing high fiber has been associated with a decrease in risk of CD but not UC.^16,17,23^

In IBD patients with undernutrition, increasing calorie intake with standard diet as well as supplemental enteral nutrition for additional calories are used as an initial approach in addition to medical therapy. In patients with a history of IBD and irritable bowel syndrome (IBS) overlap, a low FODMAP diet can reduce IBS-like symptoms and improve quality of life in patients with IBD in remission.^24^ Lactose restriction is helpful in patients with symptoms suggestive of lactose intolerance with a positive lactose breath test.

Most recently, Kane et al^25^ published new evidence-based guidelines for nutrition and diet in IBD. In patients with UC, they recommend reduction in consumption of red meat, myristic acid (palm oil, coconut oil, and dairy fats), and increasing consumption of omega-3 fatty acids but only from marine fish (not from supplements). Intake of fruits and vegetables was encouraged in CD patients without fibrostricturing disease, while saturated fats, emulsifiers, thickeners, trans fats, artificial sweeteners, unpasteurized dairy products, and processed foods containing titanium dioxide and sulfites should be avoided in both UC and CD. With regard to gluten, wheat, complex carbohydrates, refined sugars, and fructose, there was a lack of evidence to recommend limitation.

## ACCESS TO DIETITIAN CARE

It is estimated that IBD affects up to 3 million people in the United States.^26^ IBD patients have unique, individualized experiences in terms of number of flares and disease activity over time given the chronic nature of these conditions. Unfortunately, patients who have, or are at risk of malnutrition, and their physicians, face significant barriers to accessing expert and convenient nutrition care. Assessing a patient’s nutritional status, locating a registered dietitian, traveling to frequent appointments, and demographic factors such as culture and language, represent some of those challenges.

While gastroenterologists are experts in the function of the gastrointestinal tract, most are not expert in nutritional science. Most physicians receive minimal training during medical school in nutrition and the minority with the required dietary expertise frequently lack the bandwidth to meaningfully engage with patients on a longitudinal basis in order to create lasting change. Patient adherence with a nutrition plan entails frequent assessments, follow-ups, and attention to nuanced tastes and preferences.

## TELENUTRITION IN IBD

The American Gastroenterological Association (AGA) convened a multidisciplinary workgroup in December 2017 to develop a pathway to address the lack of guidance on care coordination for IBD at the system level. One of the suggested strategies for overcoming barriers and increasing access to nutrition care in IBD patients was providing nutrition therapy via telehealth.^1^ Furthermore, an assessment of 58 systematic reviews was conducted by the Agency for Healthcare Research and Quality (AHRQ), sheds light on evidence that telehealth interventions provide an effective means to address challenges related to remote monitoring and management of patients with chronic conditions.^27^ Telenutrition in IBD has the potential to improve patient satisfaction, augment outcomes, and increase access to expert dietitians with proficiency in IBD care. While telemedicine has been increasing incrementally in adoption across the United States over the past several decades, a rapid tipping point in 2020 arose in the setting of COVID-19 which has led to a surge in utilization of remote care along with increasing payer support.

While much of telemedicine has historically focused on physician and behavioral-based care, telenutrition is evolving as one of the best use cases for remote care. Given the lack of a physical examination needed in many dietitian encounters along with mobile tools that enhance the traditional brick and mortar nutrition counseling experience, telenutrition has the potential to significantly address the burden of chronic disease related to diet. Nutrimedy is a digital health company focused on medical nutrition therapy in the gastrointestinal space. Nutrimedy partners with pharmaceutical companies, health systems, physicians, and payers to create novel solutions that address chronic disease remotely such as IBD through nutritional counseling (Table 1). Given the current climate of the COVID-19 pandemic, remote nutritional counseling for chronic disease such as IBD has never been more relevant and will continue to become a cornerstone of therapy in the years to come.

**Table 1.** Nutrimedy Partners With Various Stakeholders to Improve Nutritional Therapies in IBD Patients

Health Systems	Physicians	Payers	Pharmaceutical & Medical Device Companies
Help health systems close the nutrition care gap by providing access to dietitians, education, and monitoring for any patient population.	Provide physicians an avenue for delegating longitudinal dietary care to nutrition science experts and reducing physician burden.	Partner with payers to simplify coverage and reimbursement for patients and physicians.	Enhance and differentiate device or therapy with customizable remote nutrition support and programs.

## Data Availability

No new data were created or analyzed.

## Notes

Funding: No funding was received for this review.
